# Retraction Note: Evaluation of low-cost custom made VAC therapy compared with conventional wound dressings in the treatment of non-healing lower limb ulcers in lower socio-economic group patients of Kashmir valley

**DOI:** 10.1186/s13018-017-0558-3

**Published:** 2017-04-20

**Authors:** Zameer Ali, Afshan Anjum, Lubna Khurshid, Hamayun Ahad, Saheel Maajid, Shabir Ahmad Dhar

**Affiliations:** Department of Orthopaedics SKIMS Medical College, Bemina, Srinagar, 190018 Jammu and Kashmir India

## Retraction

This article [1] has been retracted by the editor because contrary to the statement in the manuscript, the ethics committee of SKIMS Medical College have indicated that no proposal for this study was submitted to them for approval. The authors have been unable to provide documents confirming that ethics approval was obtained for the study.
